# Therapeutic Strategies Targeting Aerobic Glycolysis in Cancer and Dynamic Monitoring of Associated Metabolites

**DOI:** 10.3390/cells14161288

**Published:** 2025-08-19

**Authors:** Mengjie Hu, Kaijie Zheng, Lijiao Zhang, Yue Kan, Jiaqian Zhao, Dajing Chen

**Affiliations:** School of Pharmacy, Hangzhou Normal University, Hangzhou 311121, China; 2023112025042@stu.hznu.edu.cn (M.H.); 2024112025088@stu.hznu.edu.cn (K.Z.); 2024112025070@stu.hznu.edu.cn (L.Z.); 2023112025052@stu.hznu.edu.cn (Y.K.)

**Keywords:** metabolic reprogramming, aerobic glycolysis, metabolic biomarkers, targeted therapy, electrochemical biosensor, fluorescent biosensor

## Abstract

Cancer cells predominantly utilize aerobic glycolysis for energy production, preferentially converting glucose (Glu) to pyruvate (PA) and subsequently to lactate (LA). This metabolic reprogramming results in extracellular LA accumulation, acidifying the tumor microenvironment (TME) and facilitating tumor invasion and metastasis. The dynamics of Glu, PA, and LA are pivotal to tumor initiation and progression. This review comprehensively discussed therapeutic strategies targeting these key metabolites and systematically evaluates electrochemical and fluorescence-based techniques for their dynamic monitoring. We highlight the critical role of these monitoring approaches in advancing early cancer diagnosis, enabling personalized treatment, and accelerating anticancer drug development.

## 1. Introduction

Cancer encompasses a diverse spectrum of complex diseases characterized by the uncontrolled proliferation of genetically altered or mutated cells. A defining feature of cancer cells is their rapid growth, frequent division, and uncontrolled proliferation. Unlike normal cells that maintain tightly regulated metabolic pathways, cancer cells harbor genetic mutations that lead to the expression of dysfunctional proteins. These aberrant proteins disrupt intracellular signaling pathways and drive metabolic reprogramming [1]. By rewiring energy metabolism, cancer cells disrupt metabolic homeostasis to support rapid proliferation. This metabolic shift also promotes their invasive and metastatic potential [2].

Under physiologic conditions, normal cells preferentially generate adenosine triphosphate (ATP) via mitochondrial oxidative phosphorylation (OXPHOS), a highly efficient process fueled by the oxidation of glucose in the presence of oxygen. In contrast, hypoxic environments induce a metabolic shift towards anaerobic glycolysis for energy production. However, cancer cells exhibit a distinct metabolic preference in which they preferentially utilize glycolysis for energy production even under oxygen-rich conditions. This phenomenon, first described by Otto Warburg, is known as the ‘Warburg effect’ or aerobic glycolysis [3,4].

**Figure 1:**
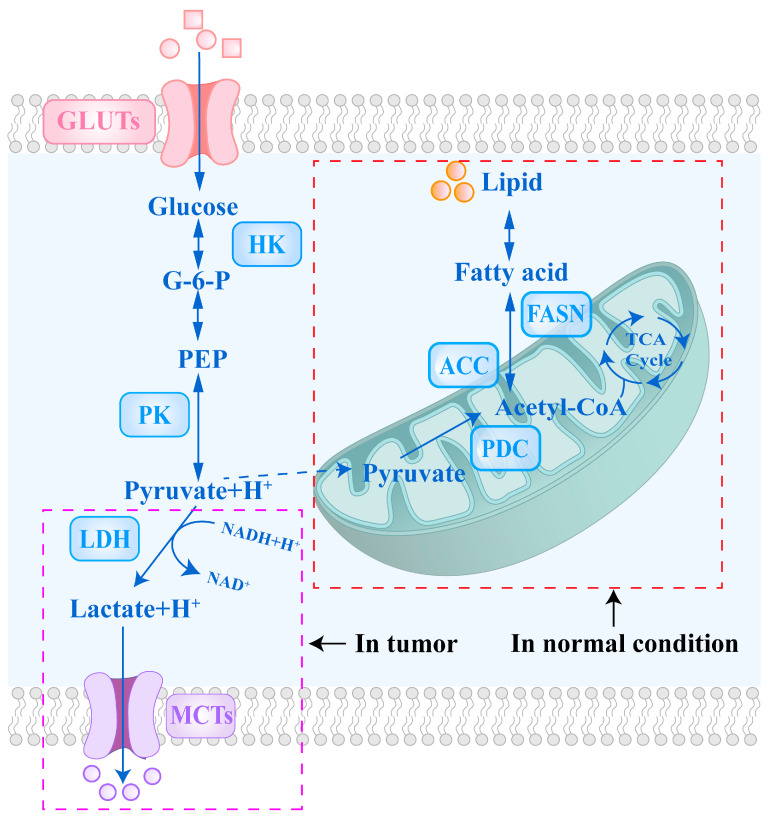
Comparison of Glycolytic Pathways in Normal and Cancer Cells. This figure illustrates the differences in glycolytic flux between normal and cancer cells, which represent a key feature of metabolic reprogramming in cancer. To meet increased anabolic and energetic demands, cancer cells significantly enhance glycolytic flux by upregulating key glycolytic enzymes and transporters. Abbreviations: GLUTs, glucose transporters; G-6-P, glucose-6-phosphate; PEP, phosphoenolpyruvate; HK, hexokinase; PK, pyruvate kinase; LDH, lactate dehydrogenase; NAD^+^, oxidized nicotinamide adenine dinucleotide; NADH, reduced nicotinamide adenine dinucleotide; PDC, pyruvate dehydrogenase complex; TCA, tricarboxylic acid; MCTs, monocarboxylate transporters; ACC, acetyl-coenzyme A carboxylase; FASN, fatty acid synthase.

As illustrated in Figure 1, aerobic glycolysis enables tumor cells to convert a substantial portion of PA into LA via lactate dehydrogenase (LDH) even under oxygen-rich conditions. The increased generation of LA facilitates the regeneration of oxidized nicotinamide adenine dinucleotide (NAD^+^), thereby sustaining continuous glycolytic flux [5,6]. However, it is important to note that metabolic phenotypes exhibit high heterogeneity both among different tumor types and within the same tumor. The degree of glycolytic activation and its impact on energy metabolism can vary significantly [7].

Alterations in tumor glucose metabolism biomarkers such as Glu, PA, and LA significantly impact the TME and immune cell activity, thereby facilitating tumor growth and progression [8]. Studies have shown that intracellular glucose depletion coupled with extracellular LA accumulation synergistically enhances tumor invasiveness, particularly correlating with high rates of distant metastasis in early tumor stages. Therefore, the dynamic fluctuations of metabolites such as Glu, PA, and LA within tumor cells and the TME serve as critical indicators of tumor metabolism and malignancy [9]. Real-time or continuous monitoring of these key metabolites offers profound insights into tumor development and provides more data for early diagnosis, prognosis evaluation, and targeted therapy, thereby advancing personalized cancer treatment and new drug development.

**Figure 2:**
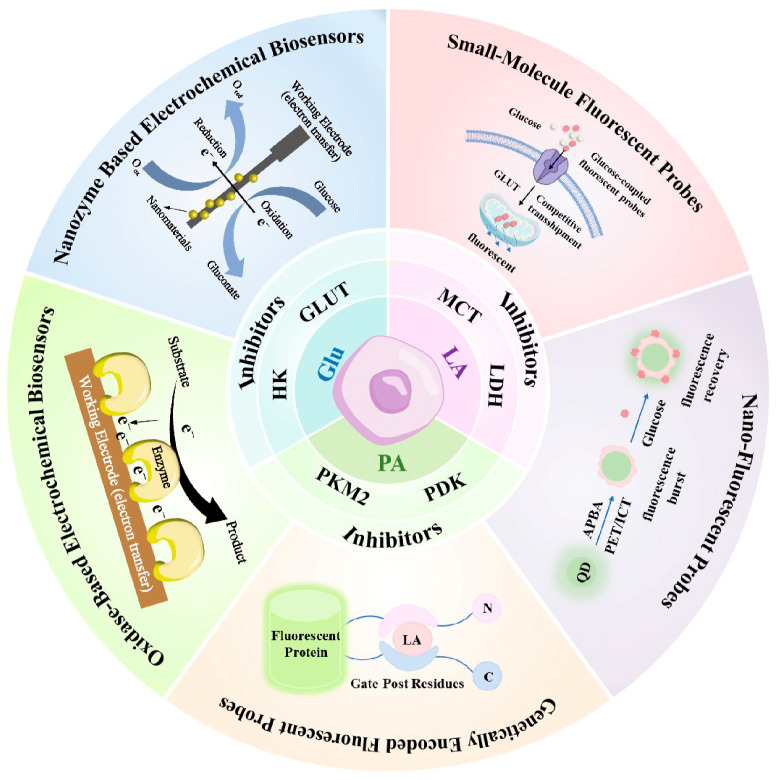
Overview of key glycolytic metabolites and their regulation in tumor metabolism, associated therapeutic targets, and representative detection technologies for dynamic monitoring at the cellular level.

This review focuses on the dynamic changes in key glycolytic metabolites—Glu, PA, and LA—in tumor cells, systematically exploring their roles in metabolic reprogramming, TME modulation, and disease progression. This review summarizes various therapeutic strategies and drugs targeting glycolysis, assessing their potential to inhibit metabolic abnormalities and impede tumor development. Importantly, this review emphasizes the critical importance of dynamic monitoring of these metabolites (Figure 2). It highlights the distinct advantages of electrochemical and fluorescence techniques for achieving high-sensitivity, non-invasive, dynamic detection at the cellular level. This work aims to provide theoretical and technical support for understanding tumor metabolism mechanisms and advancing early diagnosis, personalized therapy, and targeted drug development.

## 2. The Mechanisms of Key Glycolytic Enzymes and Transport Proteins in Tumor Cells

Glycolysis is the primary pathway through which tumor cells obtain energy and biosynthetic precursors, activated by the coordinated regulation of multiple key enzymes and transporters. Glucose enters tumor cells via glucose transporters (GLUTs), which are commonly upregulated in various cancers. Once inside the cell, glucose is phosphorylated by hexokinase (HK), especially the highly expressed HK2, to glucose-6-phosphate (G6P), initiating glycolysis and preventing glucose efflux. G6P undergoes multiple enzymatic reactions to produce key intermediates, including 3-phosphoglycerate (3-PG) and phosphoenolpyruvate (PEP), which serve as precursors for energy production and biosynthesis. Pyruvate kinase (PK) catalyzes the rate-limiting step of glycolysis, converting PEP to pyruvate and generating ATP. The low-activity dimeric form of PK promotes the accumulation of metabolic intermediates to meet cellular biosynthetic demands [10,11]. The fate of pyruvate primarily involves mitochondrial import or cytosolic reduction to lactate. Pyruvate is transported into the mitochondrial matrix via the mitochondrial pyruvate carrier (MPC) to fuel the tricarboxylic acid (TCA) cycle and support aerobic metabolism. Under hypoxic conditions or glycolysis-dominant states, pyruvate metabolism is regulated by pyruvate dehydrogenase kinase (PDK). PDK phosphorylates and inhibits the pyruvate dehydrogenase complex (PDC), blocking pyruvate entry into the TCA cycle. This inhibition promotes the reduction in pyruvate to lactate in the cytosol by lactate dehydrogenase enzyme, concurrently regenerating NAD^+^ to sustain glycolytic flux. Lactate is exported extracellularly via monocarboxylate transporters (MCTs), maintaining intracellular pH homeostasis. Furthermore, lactate accumulation acidifies the TME, enhancing tumor immune evasion and invasive potential [12].

## 3. Targeted Therapeutic Strategies for Tumor Glucose Metabolic Pathways

Glucose metabolism is fundamental to sustaining the rapid proliferation and survival of tumor cells. Despite adequate oxygen availability, tumor cells undergo metabolic reprogramming that favors aerobic glycolysis. The initial phase of this altered metabolism is characterized by enhanced glucose uptake, driven primarily by the overexpression of GLUTs [13]. The first step of aerobic glycolysis involves the conversion of Glu to PA, generating reduced nicotinamide adenine dinucleotide (NADH) and a net gain of two ATP molecules [14,15]. Beyond energy supply, glycolysis provides essential biosynthetic precursors, such as G6P for the pentose phosphate pathway and 3-PG for amino acid synthesis. Additionally, glycolysis contributes to maintaining redox homeostasis by regulating the NAD^+^/NADH ratio, thereby ensuring metabolic balance and cellular function [16]. Although aerobic glycolysis yields fewer ATP molecules per glucose molecule compared to OXPHOS, its ATP generation rate significantly surpasses that of OXPHOS. Additionally, glycolysis provides abundant biosynthetic precursors, which critically support the biosynthetic demands associated with rapid tumor proliferation [17,18]. Importantly, the upregulation of glucose metabolism in tumors presents not only a diagnostic marker but also an attractive therapeutic target for cancer treatment [19].

### 3.1. GLUTs-Targeted Therapeutic Agents

GLUT-mediated transmembrane transport of glucose constitutes the initial and rate-limiting step of glycolysis [20]. Encoded by the solute carrier 2A gene family, GLUTs function as facilitative unidirectional transporters, moving glucose along its concentration gradient. Notably, overexpression of GLUT1 and GLUT3 is frequently observed across various cancer types [21,22]. Due to their critical role in glucose uptake, GLUTs have emerged as highly attractive targets for anticancer therapy, including natural products, natural product analogs, and synthetic small-molecule inhibitors [23]. These inhibitors suppress tumor cell proliferation primarily by blocking glucose uptake, thereby depleting intracellular glucose levels and impairing energy metabolism. Natural products have emerged as a significant and growing source of potential anticancer agents. Compounds such as curcumin [24], quercetin [25], resveratrol [26], and apigenin [27] have been identified as potential GLUT inhibitors.

The rational design of novel small-molecule GLUT inhibitors constitutes another promising therapeutic strategy aimed at reducing glucose levels in cancer cells. WZB117 is a synthetic GLUT1 inhibitor that blocks glucose uptake and suppresses tumor cell proliferation. Its combination with apatinib exhibits synergistic activity in melanoma, significantly reducing both intracellular glucose and lactate production [28,29]. Moreover, WZB117 exhibits as a radiosensitizer, enhancing tumor cell response to radiotherapy. However, its clinical translation is limited by the instability of its ester bond under aqueous physiological conditions and its selectivity exclusively toward GLUT1 [30]. Similarly, Miller et al. reported BAY-876, a high-affinity GLUT1 inhibitor (IC_50_ ≈ 2 nM), which potently suppresses glucose uptake and cellular viability in head and neck squamous cell carcinoma (HNSCC) cells [31].

The design of lead compounds based on natural product fragments offers a novel approach to the development of GLUT inhibitors. Ceballos et al. introduced the concept of pseudo-natural products and developed Glupin, a compound that selectively inhibits GLUT1 and GLUT3, effectively reducing intracellular glucose levels and suppressing cancer cell growth [32]. Similarly, Karageorgis et al. designed chromopynone by fusing chromane and tetrahydropyrimidinone fragments, exhibiting potent inhibitory activity against colorectal adenocarcinoma cells [33].

### 3.2. HK-Targeted Therapeutic Agents

HK catalyzes the critical first step of glucose metabolism, phosphorylating glucose to G6P. Among the five human HK isoforms been identified, HK2 is frequently overexpressed in diverse cancer cells and largely absent in normal tissues, making it a promising selective target for cancer therapy [34]. Reported HK2 inhibitors include metformin (Met), 2-deoxyglucose (2-DG), and 3-bromopyruvate (3-BrPA). However, Met exhibits only weak antitumor activity both in vitro and in vivo as a HK2 inhibitor. Moreover, high doses of 2-DG may induce neurotoxic effects while inhibiting glycolysis in cancer cells [35], and 3-BrPA’s clinical potential is limited by dose-dependent toxicity [36].

Benserazide (BENZ) and benitrobenrazide (BNBZ) are hydrazone derivatives that share a common 2,3,4-trihydroxybenzaldehyde moiety, which mimics the glucose ring and completely occupies the glucose-binding site of HK2 [37,38]. Zheng et al. demonstrated that BNBZ inhibits HK2 (IC_50_ = 0.53 ± 0.13 μM), reducing cellular glucose metabolism and intracellular glucose levels. In addition to inhibiting glycolytic flux, BNBZ significantly suppresses serine–glycine-associated amino acid biosynthesis by reducing the production of G6P and one-carbon donors, thereby further exacerbating metabolic reprogramming in cancer cells [39]. Li et al. reported BENZ exhibits higher selectivity toward HK2, with an IC_50_ of 5.52 ± 0.17 μM, and significantly reduces glucose uptake in SW480 cells compared to metformin-treated controls [40]. Beyond direct inhibitors, certain natural products can modulate HK2 expression or activity indirectly. Identified natural compounds with significant HK2 inhibitory effects include tylophorine [41], xanthohumol [42,43], chrysin [44], matrine [45], daurisoline [46], and kaempferol [47]. These findings provide important clues for developing novel anticancer drugs based on glycolysis inhibition.

Although current representative HK2 inhibitors demonstrate tumor growth suppression and enhance chemotherapy efficacy in animal models, their clinical translation remains significantly limited. These inhibitors commonly exhibit systemic toxicity and lack cancer cell-specific targeting, resulting in pronounced off-target effects and limited therapeutic benefit. Future efforts should focus on improving the selectivity of HK2 inhibitors and exploring their synergy with chemotherapeutic agents such as doxorubicin and paclitaxel, as well as novel metabolic inhibitors, to advance their application in combination therapies [48].

## 4. Targeted Therapeutic Strategies for Tumor Pyruvate Metabolic Pathways

In glycolysis, PK catalyzes the conversion of PEP to pyruvate, generating one molecule of ATP. Pyruvate serves as a key metabolic node, linking cytoplasmic and mitochondrial pathways in tumor cells. It can be utilized in biosynthetic reactions, reduced to lactate by LDH, and exported from the cell via MCTs or transported into the mitochondrial matrix by the MPC. Within mitochondria, PDC oxidizes pyruvate to acetyl-coenzyme A (Ac-CoA), fueling the TCA cycle to generate ATP, reducing equivalents, and biosynthetic precursors [49]. Tumor cells enhance glycolytic flux, accumulating lactate by increasing pyruvate production and redirecting its utilization. This metabolic reprogramming supports rapid proliferation, adaptation to hypoxia, and contributes to drug resistance, invasiveness, and metastasis. Consequently, targeting pyruvate metabolism represents a promising therapeutic strategy.

### 4.1. PKM2-Targeted Therapeutic Agents

Pyruvate is produced through glycolysis via an irreversible phosphorylation reaction catalyzed by PK. Among the four PK isoforms, PKM2 is predominantly expressed in highly proliferative cells, including cancers. PKM2 promotes the accumulation of glycolytic intermediates that support tumor growth [50]. Due to its upregulation across various tumor types, PKM2 is regarded as a promising therapeutic target. Several small molecules targeting PKM2 have been developed to inhibit tumor growth. Tetrameric PKM2 favors complete glucose oxidation via OXPHOS, while the dimer facilitates the Warburg effect [51]. Therefore, some studies suggest that PKM2 activators can stabilize the tetrameric form of PKM2, thereby inhibiting tumor initiation and progression [52]. So far, several PKM2 tetramer activators have been reported, including N,N′-diarylsulfonamide (DASA-58) and [3,2-b]pyrrole [3,2-d]pyridazinones (TEPP-46) [53].

Conversely, PKM2 inhibitors suppress glycolysis and cancer cell proliferation by downregulating expression or inhibiting dimeric PKM2. The natural compound shikonin inhibits PKM2, reducing glucose uptake and lactate production, ultimately inducing tumor cell death [54,55,56]. BENZ directly binds and selectively inhibits PKM2 activity, suppressing aerobic glycolysis and melanoma cell proliferation, invasion, and metastasis [57]. Dimitrijevs et al. developed a series of isomer-selective PKM2 inhibitors containing a carbonyl-[1,2]isothiazole[2,3-a]pyridinium moiety. These inhibitors were found to reduce the proportion of PKM2 dimers and induce unstable conformations. The most active compound exhibited an IC_50_ value of 0.35 ± 0.07 μM [58].

### 4.2. PDK-Targeted Therapeutic Agents

In mitochondria, pyruvate undergoes oxidative decarboxylation by the PDC to generate Ac-CoA, carbon dioxide, and NADH. NADH enters the electron transport chain and drives ATP synthesis via OXPHOS, meeting cellular energy demands [59,60]. PDC activity is negatively regulated by PDK [61]. Cancer metabolic reprogramming frequently upregulates PDK, making PDK inhibition to restore PDC activity a promising therapeutic strategy [59].

**Figure 3:**
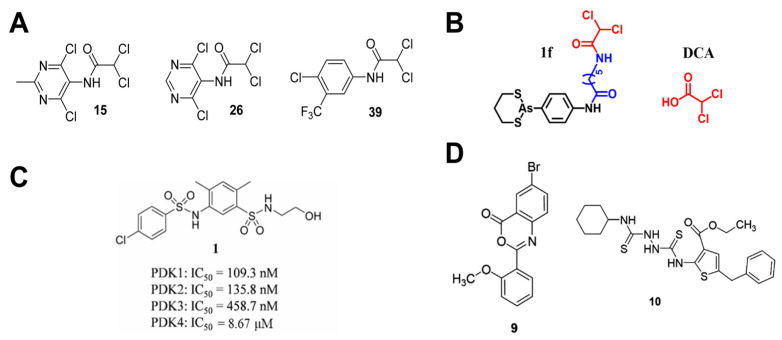
PDK inhibitors: (**A**) Structures of compounds **15**, **26** and **39** (adapted from [64]); (**B**) Structures of compound **1f** and DCA (adapted from [65]); (**C**) Structures and activities of compounds **1** (adapted from [66]); (**D**) Structures of compounds **9** and **10** (adapted from [67]).

The PDK family comprises four mitochondrial matrix isoforms (PDK1-4) sharing ~70% sequence homology [62]. To date, several PDK inhibitors have been developed, including the pyruvate analog dichloroacetate (DCA), which binds the pyruvate site. However, DCA’s low binding affinity requires high therapeutic doses, and it exhibits significant neurotoxicity [63]. Based on DCA, Zhang et al. synthesized analogs (e.g., compounds **15**, **26**, and **39**, Figure 3A) that inhibit cancer cell proliferation at micromolar levels and reprogram glucose metabolism from lactate production toward OXPHOS [64]. She et al. designed and synthesized hybrid DCA-arsenic inhibitors, with compound **1f** (Figure 3B) exhibiting superior PDK inhibition versus DCA at cellular and enzymatic levels. Compound **1f** also significantly affects alanine–aspartate–glutamate metabolism and central carbon metabolism in cancer cells. These pathways are directly involved in cellular carbon utilization and ATP production, indicating that **1f** effectively disrupts the fundamental energy metabolism of tumor cells [65].

Recent compound library screening identified more PDK inhibitors with anticancer potential. Structure-based virtual screening yielded a PDK1-3 inhibitor (compound **1**, Figure 3C) that suppressed A549 cell proliferation, invasion, migration, and induced apoptosis [66]. Additionally, Zhang et al. developed a high-throughput screening system based on the ChemBridge compound library and identified compounds **9** and **10** (Figure 3D) as micromolar level PDK1 inhibitors that restored mitochondrial function, suppressed glycolysis, and blocked proliferation [67]. Plant-derived natural products also demonstrate PDK inhibitory potential. Quercetin [68,69], thymol [70], and thymoquinone [71] exhibit strong PDK3 affinity and anticancer effects.

## 5. Targeted Therapeutic Strategies for Tumor Lactate Metabolic Pathways

In aerobic glycolysis, glucose is primarily metabolized to pyruvate, which is rapidly reduced to LA by LDH as the terminal glycolytic product [72]. Tumor cells exhibit excessive LA production and efflux, driven by hypoxia-inducible factor-1α (HIF-1α) and cellular Myc upregulation, which promotes aberrant expression of glycolytic enzymes and MCTs [73]. HIF-1α is activated under hypoxic conditions and induces the expression of key glycolytic enzymes such as hexokinase II (HKII), phosphofructokinase 1 (PFK1), LDHA, and GLUT1/3, thereby promoting glucose metabolic reprogramming. Additionally, HIF-1α transcriptionally activates PDK1 and MAX-interacting protein 1 (MXI1), which inhibit mitochondrial oxidative phosphorylation and enhance glycolytic dependency. As another key transcription factor, c-Myc promotes glucose uptake and metabolism by upregulating multiple glycolysis-related genes. It also supports glycolysis by activating glutamine metabolism to supply carbon sources [74] MCT overexpression accelerates LA secretion from the cytosol to the extracellular microenvironment [75]. In addition, multiple signaling pathways are aberrantly activated in the regulation of tumor metabolism, among which the PI3K/AKT/mTOR pathway is particularly critical. This pathway enhances the activity of key glycolytic enzymes, promoting glucose uptake and lactate production in cancer cells [76]. High levels of LA lower the pH of the TME, fostering angiogenesis, proteolytic activity, metastasis, and drug resistance. Recent studies have uncovered metabolic symbiosis within tumors, where hypoxic cells produce large amounts of LA via aerobic glycolysis, and adjacent normoxic cells use this LA as an oxidative fuel through the LA shuttle, enabling metabolic cooperation [77]. This metabolic heterogeneity not only maintains the metabolic flexibility of tumor cells but also contributes to LA accumulation in the TME. LA accumulation suppresses effector immune cells, promotes immunosuppressive cell populations, inhibits antitumor immune responses, and accelerates tumor progression, ultimately resulting in poor prognosis [78,79]. Therefore, as a critical byproduct of metabolic reprogramming in tumors, LA and its associated metabolic enzymes and transporters have emerged as promising therapeutic targets for cancer treatment.

### 5.1. LDH-Targeted Therapeutic Agents

LDH, an oxidoreductase composed of LDHA and LDHB subunits, catalyzes glycolytic redox reactions. LDHA primarily converts pyruvate to lactate, while LDHB catalyzes the reverse reaction [80]. LDHA inhibitors are classified by their mechanism: pyruvate substrate-competitive, NADH cofactor-competitive, or dual-competitive [81]. Oxalate, a pyruvate analog, acts as a substrate-competitive LDHA inhibitor validated in vitro. It suppresses brain tumor cell proliferation, invasion, and lactate production [82,83,84]. However, its high polarity and poor membrane permeability necessitate excessively high concentrations, preventing in vivo application and clinical translation [85]. Kim et al. identified selenophenyl compounds that inhibit LDHA activity. Among them, 1-(phenylseleno)-4-(trifluoromethyl) benzene (PSTMB) exhibited the strongest activity and significantly reduced LA production under hypoxia. Notably, the IC_50_ of PSTMB (145.2 nM) is much lower than that of oxalate (130.6 μM) [86].

NADH-competitive LDHA inhibitors include FX11 and derivatives of quinoline-3-sulfonamides [87]. FX11, a derivative of gossypol, exhibits selective inhibitation towards LDHA [88]. It shows preclinical efficacy in breast cancer and prostate cancer [89,90]. However, studies have shown that although FX11 exhibits some in vivo activity, its limited selectivity toward LDH and pronounced off-target effects hinder further application [91]. Quinoline-3-sulfonamides demonstrate high LDHA selectivity and activity in hepatocellular carcinoma and breast cancer [92].

Dual-competitive inhibitors, such as N-hydroxyindole (NHI) derivatives, target both substrate and cofactor sites [93,94]. NHI-sugar conjugates exhibit potent antiproliferative activity against lung and cervical cancer cells, reducing lactate levels by over 50% [95]. Several peptides as competitive LDHA inhibitors are designed to target both the NADH and pyruvate binding sites. The study revealed that KVVYNVA and IYNLLK peptides significantly decreased lactate production in cancer cells [96]. Despite promising preclinical anticancer activity, no LDHA inhibitor has advanced to clinical trials. Future efforts must prioritize addressing safety and selectivity to develop viable candidates.

Although small-molecule LDHA inhibitors have demonstrated significant antitumor potential in preclinical studies, no true clinical benefits have been observed thus far. To date, only one phase III clinical trial (NCT01977209) has evaluated the efficacy of gossypol in combination with docetaxel and cisplatin in patients with advanced non-small cell lung cancer characterized by high expression of apurinic/apyrimidinic endonuclease 1 [97]. Future efforts should focus on addressing key issues such as drug safety to facilitate the development of more effective and less toxic LDHA inhibitors.

### 5.2. MCTs-Targeted Therapeutic Agents

LA, a hydrophilic weak acid, requires MCTs for transmembrane transport. Encoded by solute carrier family 16 genes, MCT1-4 facilitate proton-linked monocarboxylate transport, with directionality determined by proton gradients and substrate concentrations [98,99]. LA efflux via MCT1 and MCT4 acidifies the TME, suppresses effector T and natural killer cell functions, sustains regulatory T cell immunosuppression, and stimulates tumor-associated macrophages to release pro-tumor factors. Together, these actions promote immune evasion and reduce immunotherapy efficacy [100]. Thus, MCT-mediated LA efflux is both a crucial metabolic regulator and a key driver of tumor immune escape. Current MCT inhibitors, including stilbene sulfonates, quercetin, phloretin, lack selectivity and affect off-target proteins [101]. Hence, the development of selective and potent MCT1 and MCT4 inhibitors is urgently needed.

AR-C155858 (AstraZeneca) is a potent nanomolar-range MCT1 inhibitor demonstrating anticancer efficacy in multiple myeloma and breast cancer models [102,103]. Its orally bioavailable analog, AZD3965, exhibits comparable potency and specificity, with preferential MCT1 inhibition. Moreover, AZD3965 significantly reduces phosphocholine levels, indicating its regulation of phospholipid metabolism, which in turn affects cell membrane composition and the balance of related metabolic intermediates [104]. AZD3965 is currently in Phase I trials for advanced solid tumors and lymphomas (NCT01791595) [105,106,107]. Moreover, increasing attention has been given to the development of MCT4 inhibitors. The triazolopyrimidine AZD0095 potently inhibits proliferation in MCT4-overexpressing tumor cells, suppressing lactate export with minimal effect on cells with high MCT1 expression [108].

Besides selective inhibitors, dual MCT1/MCT4 inhibitors such as NGY-B have demonstrated promising efficacy in the treatment of melanoma and triple-negative breast cancer [109,110]. Syrosingopine, a derivative of reserpine, also demonstrates notable anticancer activity combined with metformin across various cancer types [111]. These inhibitors disrupt lactate transport, reducing intracellular lactate accumulation and TME acidification, thereby suppressing tumor metabolic adaptation and invasion.

MCT1 and MCT4 play critical roles in lactate metabolism and tumor immunity, making combined inhibition strategies increasingly important. Currently, programmed cell death protein 1 (PD-1) immunotherapy is a standard treatment for various cancers. However, lactate transported via MCT1/MCT4 in the TME induces a dual immunosuppressive mechanism in immune cells, leading to therapeutic resistance [112]. Studies show that inhibiting MCTs can reverse immune suppression and synergize with anti-PD-1 therapy, demonstrating promising clinical potential.

## 6. Dynamic Monitoring of Metabolic Biomarkers

Disruptions in energy metabolism is characterized by enhanced glucose uptake, accumulation of pyruvate that escapes mitochondrial oxidative metabolism, and excessive lactate secretion. Notably, alterations in key glycolytic intermediates, including Glu, PA, and LA, exhibit significant potential as cancer diagnostic biomarkers [113,114]. The concentrations of these metabolic biomarkers within tumors and the TME dynamically change with tumor progression, metabolic activity, and therapy. Cells are the fundamental units of metabolic regulation, and significant metabolic heterogeneity exists among different tumor cells; therefore, sensing and monitoring metabolites at the cellular level is crucial for accurately assessing tumor metabolic states and therapeutic responses. Metabolite variations serve as critical indicators of tumor biology and inform pharmacodynamic assessment and targeted therapeutic strategies. Many anticancer agents act by modulating specific metabolite levels, highlighting the clinical value of real-time monitoring systems for timely efficacy assessment and personalized treatment optimization. Current monitoring methods include spectroscopy, mass spectrometry, electrochemical detection, and positron emission tomography (PET) [115]. PET indirectly reflects tumor glucose metabolism by imaging the radioactive glucose analog fluorine-18 fluorodeoxyglucose but has limited resolution, cannot track downstream metabolites, and involves radiation risks, restricting its widespread use [19]. Mass spectrometry offers high sensitivity and broad detection range, making it a key technique for analyzing cellular metabolic heterogeneity; however, complex sample preparation limits its real-time application [116]. Spectroscopy provides rapid and sensitive detection suitable for simultaneous multi-component analysis, but nuclear magnetic resonance (NMR) spectroscopy has low sensitivity and high equipment costs, while Raman spectroscopy suffers from weak signals and susceptibility to interference [117]. Fluorescence and electrochemical methods are particularly promising for real-time cellular monitoring due to their high sensitivity, rapid response, and operational simplicity [118,119].

### 6.1. Electrochemical Methods

Electrochemical methods offer a powerful platform for cancer diagnostics, enabling real-time, highly sensitive detection of electrochemical signals arise from biorecognition at the electrode interface [120]. Electrochemical biosensors are extensively studied for their low cost, ease of fabrication and miniaturization, scalability, continuous monitoring of cancer biomarker dynamics and chemotherapy efficacy [121]. Advances in nanomaterials, interface modification, and microfluidics are expected to further enhance their sensitivity and selectivity.

#### 6.1.1. Oxidase-Based Electrochemical Biosensors

Enzymes are biological catalysts that accelerate reaction rates by lowering activation energy with high substrate specificity [122]. Oxidase-based electrochemical biosensors quantify analytes via electrical signals generated by redox-active species during catalysis. Enzymes immobilized on electrode surfaces mediate redox processes, producing signals proportional to analyte concentration [123]. Nanomaterials serve as ideal enzyme carriers due to their high surface area and favorable physicochemical properties, increasing enzyme loading and facilitating electron transfer between enzymes and electrodes [124]. Nanocarriers can be categorized by material properties into inorganic materials, organic polymers, carbon-based materials, and hybrid composites.

**Figure 4:**
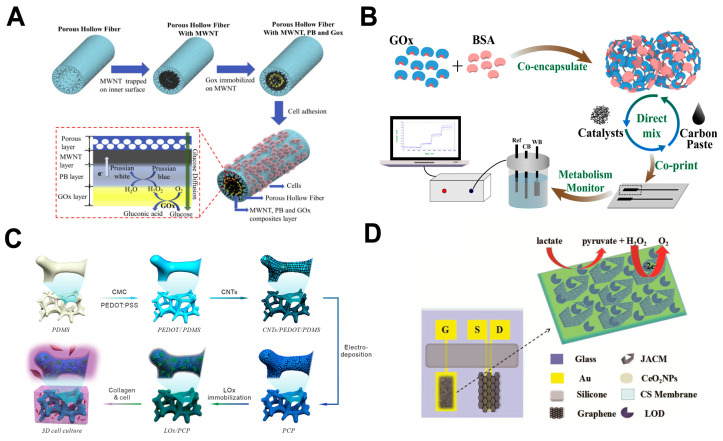
Oxidase-based electrochemical biosensors: (**A**) Schematic illustration of the PHF electrode structure and sensing principle of the electrochemical reaction catalyzed by GOx and PB (adapted from [126]); (**B**) Manufacturing process and detection schematic diagram of GOx MPs sensors (adapted from [129]); (**C**) Schematic of the fabrication processes of the 3D electrochemical sensor and collagen hydrogel integrated platform (adapted from [133]); (**D**) The schematic diagram of a solution-gated graphene transistor (adapted from [135]).

Organic polymer matrices are widely used for stable enzyme immobilization on electrodes [125]. Ma et al. developed a polyethersulfone hollow fiber (PHF) with dual functions of sensing and cell culture. Human lung cancer PC9 cells were cultured on the PHF outer surface, while glucose oxidase (GOx) and nanocatalyst were immobilized within the PHF lumen (Figure 4A). This sensor enabled continuous monitoring of glucose metabolism and has been successfully applied to evaluate osimertinib on the PC9 proliferation [126]. Similarly, microneedle array electrodes were constructed by crosslinking polyterthiophene and GOx via amide bonds, enabling efficient monitoring of glucose levels in both cancer cells and normal cells [127].

Inorganic nanocarriers are extensively studied as enzyme immobilization supports due to their superior biocompatibility, electrocatalytic activity, and structural stability [128]. Wang et al. fabricated printable GOx-nanoparticle composite electrodes via a manganese carbonate co-precipitation method (Figure 4B). These electrodes exhibited high sensitivity and stability, with a glucose detection range of 0–30 mM and stable glucose monitoring period in H1299 tumor cells over two days [129]. Lin et al. developed a self-powered lactate sensor by immobilizing lactate oxidase (LOx) on a zinc oxide (ZnO) nanowire array. This sensor detected lactate secreted by 4T1 breast cancer cells with a detection limit of 1.3 mM and a linear range up to 27 mM [130].

Carbon-based nanomaterials, including carbon nanotubes (CNTs), graphene, and their derivatives, have been widely reported as enzyme immobilization supports due to their conductivity and stability [131]. Carbon materials are often combined with organic or inorganic functional materials, improving both enzyme immobilization and sensor sensitivity. For example, an electrochemical sensor utilizing a composite carrier of single-walled carbon nanotubes and palladium nanoparticles (Pd NPs) achieved efficient glucose detection in gastric cancer MGC803 cells [132]. Yao et al. constructed a three-dimensional (3D) enzymatic electrochemical sensor on a conductive CNT scaffold incorporating Prussian blue nanoparticles (PB NPs) and LOx (Figure 4C). The sensor exhibits a linear response to lactate in the range of 0.02–1 mM, with a detection limit of 0.75 μM, and can be integrated with C6 glioma cell collagen hydrogels for dynamic lactate release monitoring [133]. Mi et al. developed a microchannel biosensor by modifying electrodes with Prussian blue and applying a chitosan-LOx-CNT composite for enzyme immobilization and signal amplification, enabling sensitive monitoring of lactate metabolism and drug-response in HepG2 cells [134]. Additionally, a transistor sensor utilized three-dimensional porous carbon loaded with cerium oxide nanoparticles (JACM/CeO_2_) (Figure 4D). Covalent grafting of LOx ensured stable immobilization and signal enhancement, achieving cellular lactate detection with a detection limit of 300 nM [135].

While oxidase-based electrochemical sensors have advanced significantly in detecting Glu and LA at cellular level. However, monitoring another key metabolic intermediate, PA, remains challenging. Current methods primarily analyze biofluids and serum samples, hindering dynamic cellular-level pyruvate concentration monitoring. Therefore, developing next-generation electrochemical sensors compatible with cellular environments is crucial. Achieving high spatio-temporal resolution detection of metabolites like pyruvate will be essential for elucidating metabolic regulation mechanisms, enabling early disease diagnosis, and guiding metabolic interventions. To summarize recent progress, Table 1 lists representative oxidase-based electrochemical biosensors for detecting Glu and LA at the cellular level, as well as PA in serum.

**Table 1:** Oxidase-based electrochemical sensors for Glu, PA, and LA.

Analyte	Electrode Composition	Linear Range	LOD	Practical Analysis Sample	Monitoring Characteristics	Ref.
Glu	PHF-MWNT-PB-GOx	0–32 mM	/	PC9 cells	3-day continuous monitoring	[126]
	GOx MPs	0–30 mM	/	H1299 cells	2-day continuous monitoring	[129]
	SWNT-GOx-GA/Pd NPs-Pt	2–1000 μM	0.5 μM	MGC803 cells	intermittent monitoring	[132]
	GC/GO-Ph-AuNP/GOx	0.3–20 mM	0.3 mM	HeLa cells	intermittent monitoring	[136]
	P(VI-AA)-Os/GOx/CNE	0–4 mM	/	NG108-15 cells	intermittent monitoring	[137]
	PLL-GA-GOx/QNP	0.1–8 mM	/	MDA-MB-231; MCF7 cells	intermittent monitoring	[138]
	GCE/CNT-GR-GOx	0–21 mM	2.99 µM	MiaPaCa2 cells	intermittent monitoring	[139]
LA	LOx/ZnO NWs/Ti	0–27 mM	1.3 mM	4T1-luc cells	intermittent monitoring	[130]
	LOx/PCP	0.02–1 mM	0.75 μM	C6 glioma cells	continuous monitoring	[133]
	CS/CNTs/LOx/PB/Pt	0–1 mM	4.5 μM	HepG2 cells	intermittent monitoring	[134]
	JACM/CeO_2_ NPs/ LOx/CS	3–300 μM	300 nM	HepG2; A549; Hela cells	intermittent monitoring	[135]
	LOx/MWCNT-PEDOT: PSS/Nafion	1–10 mM	4.0 ± 5 μM	MCF-7 cells	intermittent monitoring	[140]
	LOx/Au/CNT/RGO/Pt	0.05–100 mM	2.3 μM	A549 cells	intermittent monitoring	[141]
	PS/LOx/PEDOT-HFM/Pt NPs-PANI-HFM	0.1–6 mM	10 µM	PC9 cells	intermittent monitoring	[142]
PA	POx NPs/PGE	0.001–6000 μM	0.58 µM	serum	intermittent monitoring	[143]
	POx NPs/AuE	0.01–5000 μM	0.67 µM	serum	intermittent monitoring	[144]
	POx NPs/PtE	10–5000 μM	5 µM	serum	intermittent monitoring	[145]

Abbreviations:/, the original article did not mention or report the relevant content; LOD, limit of detection; GA, glutaraldehyde; MPs, microparticles; GC, glassy carbon; GO-Ph-AuNP, gold nanoparticles decorated graphene oxide nanocomposites; CNE, carbon nanofiber electrode; P(VI-AA)-Os, poly(1-vinylimidazole-co-acrylhydrazide)-osmium; PLL, poly-l-lysine; QNP, quartz nanopipette; GCE, glassy carbon electrode; GR, graphene; PCP, Prussian Blue nanoparticles–carbon nanotubes–poly(3,4-ethylenedioxythiophene); JACM, Jerusalem artichoke carbon material; CeO_2_ NPs, cerium dioxide nanoparticles; CS, chitosan; PEDOT: PSS, poly(3,4-ethylenedioxythiophene): polystyrene sulfonate; ZnO NWs, zinc oxide nanowires; Ti, titanium; PS, polystyrene; HFM, hollow fiber membrane; PANI, polyaniline; POx, pyruvate oxidase; PGE, pencil graphite electrode; AuE, gold electrode; PtE, platinum electrode.

#### 6.1.2. Nanozyme Based Electrochemical Biosensors

Nanozymes refer to nanomaterials with enzyme-like catalytic activities, capable of performing catalytic reactions under conditions where natural enzymes are unstable. As a type of artificial enzyme, nanozymes combine the functional properties of enzymes with the physicochemical advantages of nanomaterials, and have been widely applied in the construction of biosensors [146]. Nanozyme sensors are electrochemical devices that convert chemical changes into electrical signals via direct interaction between analytes and the electrode surface materials. Compared to oxidase-based sensors, nanozyme sensors offer superior stability and streamlined fabrication processes suited for clinical applications. These sensors demonstrate insensitivity to oxygen fluctuations, improved tolerance to environmental variations, and extended operational lifetimes [147]. Continuous refinement of synthetic strategies for multifunctional, morphologically diverse nanomaterials has significantly broadened electrochemical sensor design possibilities. Materials commonly employed in the construction of nanozyme sensors include metals and their compounds, inorganic carbon materials, conductive polymers, and metal–organic frameworks [148].

Metals’ excellent electrical conductivity and thermal stability have driven the development of diverse metal-based nanozyme sensors. Their electrochemical behavior facilitates efficient electron transfer between analyte redox centers and transducer surfaces [149]. At present, noble metals such as gold (Au), platinum (Pt), and Pd, as well as transition metals like cobalt and nickel and their compounds, have been widely employed in the design of nanozyme sensors [150].

Recent studies demonstrate nanozyme sensors enabling in situ, real-time glucose monitoring at the tumor cell level, sensitively reflecting dynamic metabolic changes. AuNPs, functioning as nanozymes mimicking natural oxidases catalysis, have been widely utilized in biosensors for the detection of Glu, LA, and other biomolecules [151]. For instance, a glucose sensing system using a gold-sputtered porous film electrode enabled in situ monitoring within MCF-7 breast tumor spheroids. This system distinguished glucose uptake under varying conditions, detecting ~12% differences post-GLUT1 inhibitor treatment, highlighting its drug screening potential [152].

Nanozyme sensors also enable in situ lactate monitoring in tumor cells, allowing real-time tracking of concentration changes. Li et al. developed a platinum microneedle platform modified with Au/polydopamine nanohybrids, featuring a lactate detection limit of 50 μM with a linear range of 0.375–12 mM [153]. While nickel oxide (NiO) nanoparticles exhibit excellent electrocatalytic activity, their poor conductivity necessitates integration with materials like reduced graphene oxide (RGO) to form performance-enhancing composites. Wang et al. developed a microfluidic system using NiO/RGO-modified screen-printed electrodes for rapid, continuous lactate monitoring in MCF-7 cell culture medium, achieving a 0.1–30 mM linear range and a 0.05 mM detection limit with excellent stability [154]. Despite significant progress in in vitro metabolite detection, the application of nanozyme electrochemical sensors at the cellular level remains relatively limited. Real-time monitoring of key metabolites like Glu and LA within live-cell microenvironments using these sensors is still absent. Therefore, further research into design strategies for cellular-level nanozyme sensors is essential to advance their utility in cellular metabolism studies and disease diagnostics.

### 6.2. Fluorescence-Based Methods

Fluorescence sensing has emerged as a pivotal methodology for tracking dynamic changes in the biological environment [155]. Its high contrast and real-time imaging capabilities permit precise monitoring key physicochemical parameters and molecular components. This technique not only facilitates quantitative analysis of ions and biomolecules but also enables noninvasive, continuous monitoring of multiple parameters in tumor cells and TME. These capabilities offer comprehensive insights into complex biological processes and disease progress, driving the development of diverse fluorescent probes—including small-molecule fluorophores, fluorescent proteins, and fluorescent nanoparticles—that specifically recognize targets and generate detectable fluorescence signal change [156,157].

#### 6.2.1. Small-Molecule Fluorescent Probes

Fluorescent probes typically comprise a signaling unit and a recognition unit, emitting fluorescence upon specific wavelength excitation to visualize biomolecules and subcellular structures. These probes are widely employed in pathological tissue imaging and functional studies [158]. Small-molecule fluorescent probes, chemically synthesized fluorophores, interact with specific biological targets to generate detectable signal changes, enabling real-time monitoring and high-resolution imaging of biological processes. These probes offer advantages such as tunable structures, synthetic accessibility, ultrasensitive detection, and exceptional spatial-temporal resolution, making them indispensable tools for cellular imaging, drug screening, and disease diagnosis [159,160].

**Figure 5:**
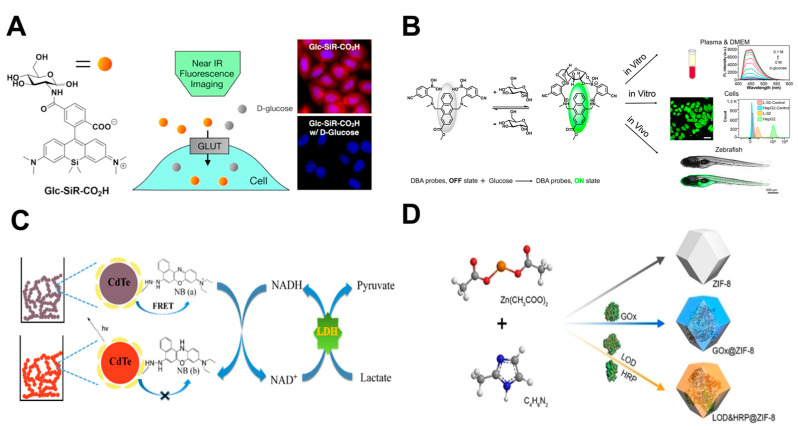
Synthesis and mechanism of fluorescent probes: (**A**) Schematic illustration of the sensing mechanism of the Glc-SiR-CO_2_H fluorescent probe (adapted from [163]); (**B**) Schematic illustration of the sensing mechanism of Mc-CDBA for glucose and its bioimaging applications (adapted from [164]); (**C**) Schematic illustration of lactate sensing by a Nile Blue-functionalized CdTe QD hydrogel (adapted from [167]); (**D**) Synthesis of ZIF-8 and enzyme@ZIF-8 (adapted from [168]).

Glucose-conjugated fluorescent probes, including fluorescently labeled 2-deoxyglucose analogs, have been widely used to monitor GLUT-mediated glucose uptake, assess anticancer drug efficacy, and investigate novel antidiabetic therapeutic strategies. Representative probes are commonly modified with fluorophores at the βC1, C2, and C6 positions of the glucose molecule [161]. Cheng et al. developed a C1-type fluorescent probe Glu-1-O-DCSN, which enters cells via GLUT1 mediation. Changes in its fluorescence intensity enable real-time monitoring of intracellular glucose uptake and utilization, highlighting the elevated glucose metabolism of tumor cells [162]. Another near-infrared fluorescent glucose tracer, Glc-SiR-CO_2_H (Figure 5A), was developed by modifying the C2 amino group of glucosamine. Its small molecular weight and strong brightness enable clear visualization of intracellular glucose uptake, permitting real-time monitoring of metabolic inhibition following anticancer drug treatment [163]. Wang et al. designed Mc-CDBA probe leveraging reversible boronate esters formation between phenyl(di)boronic acid (PDBA) and 1,2- or 1,3-diols (Figure 5B). Visualization of glucose consumption in HeLa cells showed that prolonged starvation led to decreased fluorescence intensity, indicating that the probe can sensitively monitor dynamic changes in intracellular glucose level [164].

Given pyruvate’s critical role in tumor metabolism, several small-molecule fluorescent probes have been developed for selective detection and visualization of intracellular pyruvate dynamics. Li et al. developed the 1,8-naphthalimide-based probe FPA for selective pyruvate recognition. Experiments demonstrated FPA’s capability for real-time visualization of intracellular pyruvate fluctuations during glucose-stimulated glycolysis in HeLa cells, highlighting its metabolic imaging potential [165]. Another probe, QSB, operates through Lewis acid-base reactions between diphenylboronate and pyruvate, enabling real-time fluorescent monitoring. QSB successfully tracked dynamic pyruvate levels in HeLa cells, providing direct visualization of cellular metabolic processes [166].

#### 6.2.2. Nano-Fluorescent Probes

Nano-fluorescent probes represent fluorescence-based sensors engineered from nanomaterials, offering distinct advantages including high sensitivity, target specificity, and biocompatibility. These properties have enabled their widespread application across biosensing, medical imaging, and environmental monitoring. Representative categories include quantum dots (QDs), carbon dots, and metal–organic frameworks (MOFs) [169].

Quantum dots, possessing exceptional optical properties, have emerged as valuable fluorescent nanoprobes for tumor metabolism imaging studies [170,171]. For instance, a glucose-sensitive QD-APBA probe was fabricated by covalently conjugating aminophenylboronic acid (APBA) to CdSe/ZnS QD surfaces, exhibited fluorescence lifetime increases proportional to intracellular glucose concentration [172]. Zhang et al. developed a Nile blue-modified CdTe QDs hydrogel probe with excellent biocompatibility and tunable porosity (Figure 5C). This system enabled 3D encapsulation of HeLa cells, utilizing LDH/NAD^+^ to catalyze lactate conversion into NADH, which modulated QD fluorescence for real-time lactate concentration monitoring [167].

MOFs are a class of porous crystalline materials with tunable structures and diverse properties. Zeolitic imidazolate frameworks (ZIFs), in particular, exhibit excellent crystallinity and stability, showing significant potential for applications in fluorescent sensing [173]. A dual-enzyme ZIF-8 fluorescent probe system was developed based on LOx&HRP@ZIF-8 and GOx@ZIF-8, for fluorescence detection of LA and Glu (Figure 5D). When integrated into the SingMAC chip, with stronger fluorescence signals in MCF-7 breast cancer cells indicating elevated lactate secretion and glucose uptake [168]. In addition, a multi-enzyme nano-fluorescent probe (LOx/HRP-aZIF) fabricated from amorphous ZIF permitted highly sensitive, specific intracellular lactate detection in A549 cancer cells when combined with Amplex Red [174]. Owing to their high stability and excellent intracellular biocompatibility, such MOF-based probes provide robust platforms for dynamic, continuous metabolite monitoring in living systems.

#### 6.2.3. Genetically Encoded Fluorescent Probes

Genetically encoded fluorescent probes are autonomous sensors that can be targeted to specific cells or subcellular organelles using signal peptides, enabling real-time imaging and monitoring of metabolic activities in live cells. These probes typically comprise two fundamental modules: a substrate-binding protein and a fluorescent protein. Target biomolecule interaction with the sensing protein induces conformational changes that modulate the fluorescence intensity or spectral characteristics of the fluorescent reporter [175,176].

**Figure 6:**
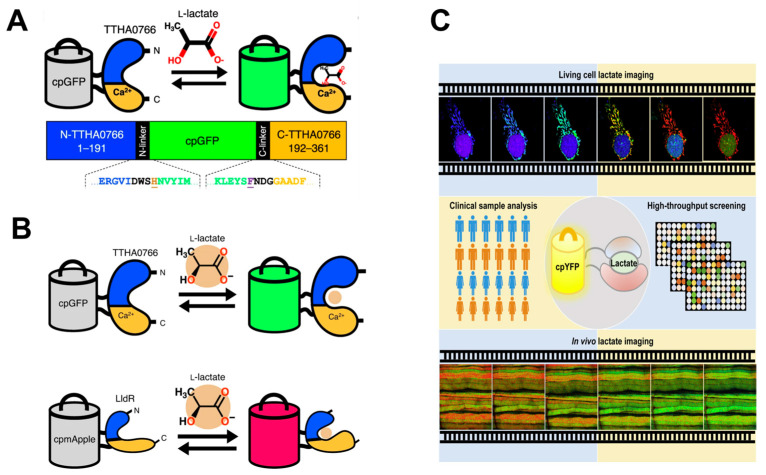
Genetically encoded lactate fluorescent probes: (**A**) Schematic representation of eLACCO1.1 and its mechanism of response to lactate (adapted from [178]); (**B**) Schematic of eLACCO2.1 and R-iLACCO1.2 response mechanisms to lactate (adapted from [179]); (**C**) Schematic illustration of the sensing mechanism of FiLa sensor and its application in lactate imaging in living cells and in vivo (adapted from [180]).

Given LA’s pleiotropic roles in physiological processes, genetically encoded lactate-specific probes have been developed as powerful tools for investigating dynamic lactate changes in cells and tissues [177]. Researchers reported the genetically encoded biosensor eLACCO1.1 (Figure 6A) for imaging endogenous lactate in glioma T98G cells. Glucose stimulation enhanced eLACCO1.1 fluorescence, while glycolysis inhibitors significantly reduced the signal, confirming the probe’s high lactate sensitivity and specificity [178]. Furthermore, spectrally and functionally orthogonal sensors were developed: the green-fluorescent eLACCO2.1 for extracellular lactate and the red-fluorescent R-iLACCO1 for intracellular lactate (Figure 6B). Co-expression in T98G cells enabled real-time monitoring of cytoplasmic lactate production and its extracellular transport [179]. In addition, the probe FiLa (Figure 6C)—comprising the lactate-sensing protein LldR embedded within a circularly permuted yellow fluorescent protein (cpYFP)—facilitated dynamic monitoring of lactate uptake, accumulation, and clearance in H1299 and HeLa cells [180].

While fluorescence detection technology enables valuable visualization of cellular metabolites, particularly for studying uptake and spatial distribution, significant limitations persist. Most probes detect only single metabolites, precluding high-throughput multiplexed detection. Fluorophore size can also interfere with downstream metabolic utilization. Challenges in sensitivity and specificity further restrict trace metabolite detection and increase vulnerability to nonspecific background fluorescence [181]. Critically, the limited variety of available metabolite-specific probes underscores the need for developing novel, highly selective, and sensitive probes to advance fluorescence-based applications in cellular metabolism research.

## 7. Discussion

Prospects: Metabolites including Glu, PA, and LA play pivotal roles in tumor metabolic reprogramming. Dynamic monitoring of these metabolites provides deeper insights into tumor metabolic characteristics and delivers crucial biochemical evidence for assessing tumor invasiveness, metastatic potential, and immune evasion. Targeting key enzymes and transport proteins to modulate metabolic states can alleviate TME acidification and improve TME homeostasis. Furthermore, as a critical technology, dynamic monitoring facilitates targeted drug screening by providing essential data for drug efficacy evaluation and lead compound optimization, thereby advancing precision medicine and personalized therapeutic strategies.

Challenges: Current monitoring techniques, including electrochemical and fluorescence methods, face persistent limitations in sensitivity, stability, and operational simplicity due to factors such as background interference, photobleaching, and electrode fouling. Concurrently, most drugs targeting aerobic glycolysis remain at preclinical stages, hindered by inconsistent efficacy, significant side effects, and poor specificity, which collectively limit clinical translation. Additionally, TME heterogeneity imposes stringent demands on both monitoring and screening technologies. Future research should prioritize optimizing detection platforms to enhance resolution and selectivity, accelerating the clinical implementation and translational progress of metabolism-targeted therapies.

## Data Availability

Data are contained within the article.

## References

[1] Gao Y., Siyu Z., Zhang X., Du Y., Ni T., Hao S. (2024). Crosstalk between metabolic and epigenetic modifications during cell carcinogenesis. iScience.

[2] Kalyanaraman B. (2017). Teaching the basics of cancer metabolism: Developing antitumor strategies by exploiting the differences between normal and cancer cell metabolism. Redox Biol..

[3] Wallrabe H., Svindrych Z., Alam S.R., Siller K.H., Wang T., Kashatus D., Hu S., Periasamy A. (2018). Segmented cell analyses to measure redox states of autofluorescent NAD(P)H, FAD & Trp in cancer cells by FLIM. Sci. Rep..

[4] Jeong H., Kim S., Hong B.-J., Lee C.-J., Kim Y.-E., Bok S., Oh J.-M., Gwak S.-H., Yoo M.Y., Lee M.S. (2019). Tumor-Associated Macrophages Enhance Tumor Hypoxia and Aerobic Glycolysis. Cancer Res..

[5] Spencer N.Y., Stanton R.C. (2019). The Warburg Effect, Lactate, and Nearly a Century of Trying to Cure Cancer. Semin. Nephrol..

[6] Elia I., Haigis M.C. (2021). Metabolites and the tumour microenvironment: From cellular mechanisms to systemic metabolism. Nat. Metab..

[7] Wu D., Zhuo L., Wang X. (2017). Metabolic reprogramming of carcinoma-associated fibroblasts and its impact on metabolic heterogeneity of tumors. Semin. Cell Dev. Biol..

[8] Liu Y., Zhao Y., Song H., Li Y., Liu Z., Ye Z., Zhao J., Wu Y., Tang J., Yao M. (2024). Metabolic reprogramming in tumor immune microenvironment: Impact on immune cell function and therapeutic implications. Cancer Lett..

[9] Su C.K., Tseng P.J., Chiu H.T., Del Vall A., Huang Y.F., Sun Y.C. (2017). Sequential enzymatic derivatization coupled with online microdialysis sampling for simultaneous profiling of mouse tumor extracellular hydrogen peroxide, lactate, and glucose. Anal. Chim. Acta.

[10] Li Z., Zhang H. (2015). Reprogramming of glucose, fatty acid and amino acid metabolism for cancer progression. Cell. Mol. Life Sci..

[11] Yu L., Chen X., Sun X., Wang L., Chen S. (2017). The Glycolytic Switch in Tumors: How Many Players Are Involved?. J. Cancer.

[12] Abdel-Wahab A.F., Mahmoud W., Al-Harizy R.M. (2019). Targeting glucose metabolism to suppress cancer progression: Prospective of anti-glycolytic cancer therapy. Pharmacol. Res..

[13] Wang X., Wang L., Hao Q., Cai M., Wang X., An W. (2024). Harnessing glucose metabolism with nanomedicine for cancer treatment. Theranostics.

[14] Ghanavat M., Shahrouzian M., Deris Zayeri Z., Banihashemi S., Kazemi S.M., Saki N. (2021). Digging deeper through glucose metabolism and its regulators in cancer and metastasis. Life Sci..

[15] Gill K.S., Fernandes P., O’Donovan T.R., McKenna S.L., Doddakula K.K., Power D.G., Soden D.M., Forde P.F. (2016). Glycolysis inhibition as a cancer treatment and its role in an anti-tumour immune response. Biochim. Biophys. Acta Rev. Cancer.

[16] Cordani M., Michetti F., Zarrabi A., Zarepour A., Rumio C., Strippoli R., Marcucci F. (2024). The role of glycolysis in tumorigenesis: From biological aspects to therapeutic opportunities. Neoplasia.

[17] Agrawal S., Singh G.K., Tiwari S. (2024). Focused starvation of tumor cells using glucose oxidase: A comprehensive review. Int. J. Biol. Macromol..

[18] Fan S., Guo J., Nie H., Xiong H., Xia Y. (2024). Aberrant Energy Metabolism in Tumors and Potential Therapeutic Targets. Genes Chromosom. Cancer.

[19] Hay N. (2016). Reprogramming glucose metabolism in cancer: Can it be exploited for cancer therapy?. Nat. Rev. Cancer.

[20] Barron C.C., Bilan P.J., Tsakiridis T., Tsiani E. (2016). Facilitative glucose transporters: Implications for cancer detection, prognosis and treatment. Metabolism.

[21] Na K.J., Choi H., Oh H.R., Kim Y.H., Lee S.B., Jung Y.J., Koh J., Park S., Lee H.J., Jeon Y.K. (2020). Reciprocal change in Glucose metabolism of Cancer and Immune Cells mediated by different Glucose Transporters predicts Immunotherapy response. Theranostics.

[22] Shriwas P., Chen X., Kinghorn A.D., Ren Y. (2019). Plant-derived glucose transport inhibitors with potential antitumor activity. Phytother. Res..

[23] Reckzeh E.S., Waldmann H. (2019). Small-Molecule Inhibition of Glucose Transporters GLUT-1–4. ChemBioChem.

[24] Gunnink L.K., Alabi O.D., Kuiper B.D., Gunnink S.M., Schuiteman S.J., Strohbehn L.E., Hamilton K.E., Wrobel K.E., Louters L.L. (2016). Curcumin directly inhibits the transport activity of GLUT1. Biochimie.

[25] Brito A.F., Ribeiro M., Abrantes A.M., Mamede A.C., Laranjo M., Casalta-Lopes J.E., Gonçalves A.C., Sarmento-Ribeiro A.B., Tralhão J.G., Botelho M.F. (2016). New Approach for Treatment of Primary Liver Tumors: The Role of Quercetin. Nutr. Cancer.

[26] Gwak H., Haegeman G., Tsang B.K., Song Y.S. (2014). Cancer-specific interruption of glucose metabolism by resveratrol is mediated through inhibition of Akt/GLUT1 axis in ovarian cancer cells. Mol. Carcinog..

[27] Wang L., Mei Z., Jin G., Liu H., Lv S., Fu R., Li M., Yao C. (2024). In situ sustained release hydrogel system delivering GLUT1 inhibitor and chemo-drug for cancer post-surgical treatment. Bioact. Mater..

[28] De A., Wadhwani A., Sauraj, Roychowdhury P., Kang J.H., Ko Y.T., Kuppusamy G. (2023). WZB117 Decorated Metformin-Carboxymethyl Chitosan Nanoparticles for Targeting Breast Cancer Metabolism. Polymers.

[29] Zhang R.-S., Li Z.-K., Liu J., Deng Y.-T., Jiang Y. (2022). WZB117 enhanced the anti-tumor effect of apatinib against melanoma via blocking STAT3/PKM2 axis. Front. Pharmacol..

[30] Temre M.K., Kumar A., Singh S.M. (2022). An appraisal of the current status of inhibition of glucose transporters as an emerging antineoplastic approach: Promising potential of new pan-GLUT inhibitors. Front. Pharmacol..

[31] Miller Z.A., Muthuswami S., Mueller A., Ma R.Z., Sywanycz S.M., Naik A., Huang L., Brody R.M., Diab A., Carey R.M. (2024). GLUT1 inhibitor BAY-876 induces apoptosis and enhances anti-cancer effects of bitter receptor agonists in head and neck squamous carcinoma cells. Cell Death Discov..

[32] Ceballos J., Schwalfenberg M., Karageorgis G., Reckzeh E.S., Sievers S., Ostermann C., Pahl A., Sellstedt M., Nowacki J., Carnero Corrales M.A. (2019). Synthesis of Indomorphan Pseudo-Natural Product Inhibitors of Glucose Transporters GLUT-1 and -3. Angew. Chem. Int. Ed. Engl..

[33] Karageorgis G., Reckzeh E.S., Ceballos J., Schwalfenberg M., Sievers S., Ostermann C., Pahl A., Ziegler S., Waldmann H. (2018). Chromopynones are pseudo natural product glucose uptake inhibitors targeting glucose transporters GLUT-1 and -3. Nat. Chem..

[34] Shan W., Zhou Y., Tam K.Y. (2022). The development of small-molecule inhibitors targeting hexokinase 2. Drug Discov. Today.

[35] Bao F., Yang K., Wu C., Gao S., Wang P., Chen L., Li H. (2018). New natural inhibitors of hexokinase 2 (HK2): Steroids from Ganoderma sinense. Fitoterapia.

[36] Pei W.-J., Zhou H., Zeng Y., Ji Q.-L., Zhang J.-J., Wang X. (2025). 3-Bromopyruvate modified with cholesterol enhances anti-hepatoma activity by inducing ferroptosis and apoptosis. J. Drug Deliv. Sci. Technol..

[37] Juszczak K., Szczepankiewicz W., Walczak K. (2024). Synthesis and Primary Activity Assay of Novel Benitrobenrazide and Benserazide Derivatives. Molecules.

[38] Juszczak K., Kubicka A., Kitel R., Dzido G., Łabieniec-Watała M., Zawadzki S., Marczak A., Walczak K., Matczak K., Tomczyk M.D. (2022). Hexokinase 2 Inhibition and Biological Effects of BNBZ and Its Derivatives: The Influence of the Number and Arrangement of Hydroxyl Groups. Int. J. Mol. Sci..

[39] Zheng M., Wu C., Yang K., Yang Y., Liu Y., Gao S., Wang Q., Li C., Chen L., Li H. (2021). Novel selective hexokinase 2 inhibitor Benitrobenrazide blocks cancer cells growth by targeting glycolysis. Pharmacol. Res..

[40] Li W., Zheng M., Wu S., Gao S., Yang M., Li Z., Min Q., Sun W., Chen L., Xiang G. (2017). Benserazide, a dopadecarboxylase inhibitor, suppresses tumor growth by targeting hexokinase 2. J. Exp. Clin. Cancer Res..

[41] Liu W., Yu X., Zhou L., Li J., Li M., Li W., Gao F. (2020). Sinomenine Inhibits Non-Small Cell Lung Cancer via Downregulation of Hexokinases II-Mediated Aerobic Glycolysis. Onco Targets Ther..

[42] Liu W., Li W., Liu H., Yu X. (2019). Xanthohumol inhibits colorectal cancer cells via downregulation of Hexokinases II-mediated glycolysis. Int. J. Biol. Sci..

[43] Yuan J., Peng G., Xiao G., Yang Z., Huang J., Liu Q., Yang Z., Liu D. (2020). Xanthohumol suppresses glioblastoma via modulation of Hexokinase 2 -mediated glycolysis. J. Cancer.

[44] Xu D., Jin J., Yu H., Zhao Z., Ma D., Zhang C., Jiang H. (2017). Chrysin inhibited tumor glycolysis and induced apoptosis in hepatocellular carcinoma by targeting hexokinase-2. J. Exp. Clin. Cancer Res..

[45] Lin G., Wu Y., Cai F., Li Z., Su S., Wang J., Cao J., Ma L. (2019). Matrine Promotes Human Myeloid Leukemia Cells Apoptosis Through Warburg Effect Mediated by Hexokinase 2. Front. Pharmacol..

[46] Tan S.M., Luo L., He Y.F., Li W., Wan X.X. (2025). Daurisoline inhibits glycolysis of lung cancer by targeting the AKT-HK2 axis. Cancer Biol. Ther..

[47] Zheng X., Pan Y., Yang G., Liu Y., Zou J., Zhao H., Yin G., Wu Y., Li X., Wei Z. (2022). Kaempferol impairs aerobic glycolysis against melanoma metastasis via inhibiting the mitochondrial binding of HK2 and VDAC1. Eur. J. Pharmacol..

[48] Martinez-Outschoorn U.E., Peiris-Pages M., Pestell R.G., Sotgia F., Lisanti M.P. (2017). Cancer metabolism: A therapeutic perspective. Nat. Rev. Clin. Oncol..

[49] Olson K.A., Schell J.C., Rutter J. (2016). Pyruvate and Metabolic Flexibility: Illuminating a Path Toward Selective Cancer Therapies. Trends Biochem. Sci..

[50] Alquraishi M., Puckett D.L., Alani D.S., Humidat A.S., Frankel V.D., Donohoe D.R., Whelan J., Bettaieb A. (2019). Pyruvate kinase M2: A simple molecule with complex functions. Free Radic. Biol. Med..

[51] Chen X., Chen S., Yu D. (2020). Protein kinase function of pyruvate kinase M2 and cancer. Cancer Cell Int..

[52] Zhu S., Guo Y., Zhang X., Liu H., Yin M., Chen X., Peng C. (2021). Pyruvate kinase M2 (PKM2) in cancer and cancer therapeutics. Cancer Lett..

[53] El-Far A.H., Al Jaouni S.K., Li X., Fu J. (2022). Cancer metabolism control by natural products: Pyruvate kinase M2 targeting therapeutics. Phytother. Res..

[54] Su Q., Luo S., Tan Q., Deng J., Zhou S., Peng M., Tao T., Yang X. (2019). The role of pyruvate kinase M2 in anticancer therapeutic treatments. Oncol. Lett..

[55] Liu T., Li S., Wu L., Yu Q., Li J., Feng J., Zhang J., Chen J., Zhou Y., Ji J. (2020). Experimental Study of Hepatocellular Carcinoma Treatment by Shikonin Through Regulating PKM2. J. Hepatocell. Carcinoma.

[56] James A.D., Richardson D.A., Oh I.W., Sritangos P., Attard T., Barrett L., Bruce J.I.E. (2020). Cutting off the fuel supply to calcium pumps in pancreatic cancer cells: Role of pyruvate kinase-M2 (PKM2). Br. J. Cancer.

[57] Zhou Y., Huang Z., Su J., Li J., Zhao S., Wu L., Zhang J., He Y., Zhang G., Tao J. (2020). Benserazide is a novel inhibitor targeting PKM2 for melanoma treatment. Int. J. Cancer.

[58] Dimitrijevs P., Makrecka-Kuka M., Bogucka A., Hyvonen M., Pantelejevs T., Arsenyan P. (2023). Development of isoselenazolium chlorides as selective pyruvate kinase isoform M2 inhibitors. Eur. J. Med. Chem..

[59] Wang X., Shen X., Yan Y., Li H. (2021). Pyruvate dehydrogenase kinases (PDKs): An overview toward clinical applications. Biosci. Rep..

[60] Anwar S., Shamsi A., Mohammad T., Islam A., Hassan M.I. (2021). Targeting pyruvate dehydrogenase kinase signaling in the development of effective cancer therapy. Biochim. Biophys. Acta Rev. Cancer.

[61] Bessho Y., Akaki T., Hara Y., Yamakawa M., Obika S., Mori G., Ubukata M., Yasue K., Nakane Y., Terasako Y. (2021). Structure-based drug design of novel and highly potent pyruvate dehydrogenase kinase inhibitors. Bioorganic Med. Chem..

[62] Woolbright B.L., Rajendran G., Harris R.A., Taylor J.A. (2019). Metabolic Flexibility in Cancer: Targeting the Pyruvate Dehydrogenase Kinase:Pyruvate Dehydrogenase Axis. Mol. Cancer Ther..

[63] Masini T., Birkaya B., van Dijk S., Mondal M., Hekelaar J., Jäger M., Terwisscha van Scheltinga A.C., Patel M.S., Hirsch A.K.H., Moman E. (2016). Furoates and thenoates inhibit pyruvate dehydrogenase kinase 2 allosterically by binding to its pyruvate regulatory site. J. Enzym. Inhib. Med. Chem..

[64] Zhang S.-L., Hu X., Zhang W., Tam K.Y. (2016). Unexpected Discovery of Dichloroacetate Derived Adenosine Triphosphate Competitors Targeting Pyruvate Dehydrogenase Kinase To Inhibit Cancer Proliferation. J. Med. Chem..

[65] She W., Liu T., Li H., Wang Z., Guo Z., Liu Y., Liu Y. (2023). Reprogramming Energy Metabolism with Synthesized PDK Inhibitors Based on Dichloroacetate Derivatives and Targeted Delivery Systems for Enhanced Cancer Therapy. J. Med. Chem..

[66] Gan L., Yang Y., Liang Z., Zhang M., He Y., Zhang S.-L. (2024). Targeting the pyruvate dehydrogenase complex/pyruvate dehydrogenase kinase (PDC/PDK) axis to discover potent PDK inhibitors through structure-based virtual screening and pharmacological evaluation. Eur. J. Med. Chem..

[67] Zhang W., Hu X., Chakravarty H., Yang Z., Tam K.Y. (2018). Identification of Novel Pyruvate Dehydrogenase Kinase 1 (PDK1) Inhibitors by Kinase Activity-Based High-Throughput Screening for Anticancer Therapeutics. ACS Comb. Sci..

[68] Chan C.-Y., Hong S.-C., Chang C.-M., Chen Y.-H., Liao P.-C., Huang C.-Y. (2023). Oral Squamous Cell Carcinoma Cells with Acquired Resistance to Erlotinib Are Sensitive to Anti-Cancer Effect of Quercetin via Pyruvate Kinase M2 (PKM2). Cells.

[69] Dahiya R., Mohammad T., Roy S., Anwar S., Gupta P., Haque A., Khan P., Kazim S.N., Islam A., Ahmad F. (2019). Investigation of inhibitory potential of quercetin to the pyruvate dehydrogenase kinase 3: Towards implications in anticancer therapy. Int. J. Biol. Macromol..

[70] Jairajpuri D.S., Khan S., Anwar S., Hussain A., Alajmi M.F., Hassan I. (2024). Investigating the role of thymol as a promising inhibitor of pyruvate dehydrogenase kinase 3 for targeted cancer therapy. Int. J. Biol. Macromol..

[71] Alotaibi B.S., Hakami M.A., Anwar S., Mawkili W., Albaqami A., Hassan M.I. (2024). Structure-based investigation of pyruvate dehydrogenase kinase-3 inhibitory potential of thymoquinone, targeting lung cancer therapy. Int. J. Biol. Macromol..

[72] Byun J.-K. (2023). Tumor lactic acid: A potential target for cancer therapy. Arch. Pharm. Res..

[73] Wang Z.-H., Peng W.-B., Zhang P., Yang X.-P., Zhou Q. (2021). Lactate in the tumour microenvironment: From immune modulation to therapy. eBioMedicine.

[74] Wu Z., Wu J., Zhao Q., Fu S., Jin J. (2020). Emerging roles of aerobic glycolysis in breast cancer. Clin. Transl. Oncol..

[75] Jiang B. (2017). Aerobic glycolysis and high level of lactate in cancer metabolism and microenvironment. Genes Dis..

[76] Varghese E., Samuel S.M., Líšková A., Samec M., Kubatka P., Büsselberg D. (2020). Targeting Glucose Metabolism to Overcome Resistance to Anticancer Chemotherapy in Breast Cancer. Cancers.

[77] Morrot A., da Fonseca L.M., Salustiano E.J., Gentile L.B., Conde L., Filardy A.A., Franklim T.N., da Costa K.M., Freire-de-Lima C.G., Freire-de-Lima L. (2018). Metabolic Symbiosis and Immunomodulation: How Tumor Cell-Derived Lactate May Disturb Innate and Adaptive Immune Responses. Front. Oncol..

[78] Lin J., Liu G., Chen L., Kwok H.F., Lin Y. (2022). Targeting lactate-related cell cycle activities for cancer therapy. Semin. Cancer Biol..

[79] Mortazavi Farsani S.S., Verma V. (2023). Lactate mediated metabolic crosstalk between cancer and immune cells and its therapeutic implications. Front. Oncol..

[80] Jafary F., Ganjalikhany M.R., Moradi A., Hemati M., Jafari S. (2019). Novel Peptide Inhibitors for Lactate Dehydrogenase A (LDHA): A Survey to Inhibit LDHA Activity via Disruption of Protein-Protein Interaction. Sci. Rep..

[81] Sharma D., Singh M., Rani R. (2022). Role of LDH in tumor glycolysis: Regulation of LDHA by small molecules for cancer therapeutics. Semin. Cancer Biol..

[82] An J., Zhang Y., He J., Zang Z., Zhou Z., Pei X., Zheng X., Zhang W., Yang H., Li S. (2017). Lactate dehydrogenase A promotes the invasion and proliferation of pituitary adenoma. Sci. Rep..

[83] Valvona C.J., Fillmore H.L. (2018). Oxamate, but Not Selective Targeting of LDH-A, Inhibits Medulloblastoma Cell Glycolysis, Growth and Motility. Brain Sci..

[84] Altinoz M.A., Ozpinar A. (2022). Oxamate targeting aggressive cancers with special emphasis to brain tumors. Biomed. Pharmacother..

[85] Feng Y., Xiong Y., Qiao T., Li X., Jia L., Han Y. (2018). Lactate dehydrogenase A: A key player in carcinogenesis and potential target in cancer therapy. Cancer Med..

[86] Kim E.Y., Chung T.W., Han C.W., Park S.Y., Park K.H., Jang S.B., Ha K.T. (2019). A Novel Lactate Dehydrogenase Inhibitor, 1-(Phenylseleno)-4-(Trifluoromethyl) Benzene, Suppresses Tumor Growth through Apoptotic Cell Death. Sci. Rep..

[87] Li Z., Cui J. (2023). Targeting the lactic acid metabolic pathway for antitumor therapy. Mol. Ther. Oncolytics.

[88] Rellinger E.J., Craig B.T., Alvarez A.L., Dusek H.L., Kim K.W., Qiao J., Chung D.H. (2017). FX11 inhibits aerobic glycolysis and growth of neuroblastoma cells. Surgery.

[89] Liu J., Zhang C., Zhang T., Chang C.Y., Wang J., Bazile L., Zhang L., Haffty B.G., Hu W., Feng Z. (2022). Metabolic enzyme LDHA activates Rac1 GTPase as a noncanonical mechanism to promote cancer. Nat. Metab..

[90] Hou X., Shi X., Zhang W., Li D., Hu L., Yang J., Zhao J., Wei S., Wei X., Ruan X. (2021). LDHA induces EMT gene transcription and regulates autophagy to promote the metastasis and tumorigenesis of papillary thyroid carcinoma. Cell Death Dis..

[91] Oshima N., Ishida R., Kishimoto S., Beebe K., Brender J.R., Yamamoto K., Urban D., Rai G., Johnson M.S., Benavides G. (2020). Dynamic Imaging of LDH Inhibition in Tumors Reveals Rapid In Vivo Metabolic Rewiring and Vulnerability to Combination Therapy. Cell Rep..

[92] Woodford M.R., Chen V.Z., Backe S.J., Gennady B., Mollapour M. (2020). Structural and Functional Regulation of Lactate dehydrogenase-A in Cancer. Future Med. Chem..

[93] Xing W., Li X., Zhou Y., Li M., Zhu M. (2023). Lactate metabolic pathway regulates tumor cell metastasis and its use as a new therapeutic target. Explor. Med..

[94] Angulo-Elizari E., Gaviria-Soteras L., Zubiri I., Ramos-Inza S., Sanmartin C., Plano D. (2023). Unmasking the Warburg Effect: Unleashing the Power of Enzyme Inhibitors for Cancer Therapy. Drugs Drug Candidates.

[95] D’Andrea F., Vagelli G., Granchi C., Guazzelli L., Tuccinardi T., Poli G., Iacopini D., Minutolo F., Di Bussolo V. (2019). Synthesis and Biological Evaluation of New Glycoconjugated LDH Inhibitors as Anticancer Agents. Molecules.

[96] Jafary F., Moradi A., Ganjalikhany M.R., Hassanpour Dehnavi A., Seifati S.M., Khodarahmi A., Hemati M. (2025). Investigation of the inhibitory peptide effect as novel strategy in cancer treatment: Targeting the tetramerization of lactate dehydrogenase A. Int. J. Biol. Macromol..

[97] Tang Y., Gu S., Zhu L., Wu Y., Zhang W., Zhao C. (2022). LDHA: The Obstacle to T cell responses against tumor. Front. Oncol..

[98] Wang N., Jiang X., Zhang S., Zhu A., Yuan Y., Xu H., Lei J., Yan C. (2021). Structural basis of human monocarboxylate transporter 1 inhibition by anti-cancer drug candidates. Cell.

[99] Payen V.L., Mina E., Van Hée V.F., Porporato P.E., Sonveaux P. (2020). Monocarboxylate transporters in cancer. Mol. Metab..

[100] Babl N., Decking S.M., Voll F., Althammer M., Sala-Hojman A., Ferretti R., Korf C., Schmidl C., Schmidleithner L., Nerb B. (2023). MCT4 blockade increases the efficacy of immune checkpoint blockade. J. Immunother. Cancer.

[101] Wu P., Zhou Y., Guo Y., Zhang S.-L., Tam K.Y. (2021). Recent developments of human monocarboxylate transporter (hMCT) inhibitors as anticancer agents. Drug Discov. Today.

[102] Nancolas B., Sessions R.B., Halestrap A.P. (2015). Identification of key binding site residues of MCT1 for AR-C155858 reveals the molecular basis of its isoform selectivity. Biochem. J..

[103] Guan X., Bryniarski M.A., Morris M.E. (2018). In Vitro and In Vivo Efficacy of the Monocarboxylate Transporter 1 Inhibitor AR-C155858 in the Murine 4T1 Breast Cancer Tumor Model. AAPS J..

[104] Beloueche-Babari M., Casals Galobart T., Delgado-Goni T., Wantuch S., Parkes H.G., Tandy D., Harker J.A., Leach M.O. (2020). Monocarboxylate transporter 1 blockade with AZD3965 inhibits lipid biosynthesis and increases tumour immune cell infiltration. Br. J. Cancer.

[105] Puri S., Juvale K. (2020). Monocarboxylate transporter 1 and 4 inhibitors as potential therapeutics for treating solid tumours: A review with structure-activity relationship insights. Eur. J. Med. Chem..

[106] Barbato A., Giallongo C., Giallongo S., Romano A., Scandura G., Concetta S., Zuppelli T., Lolicato M., Lazzarino G., Parrinello N. (2023). Lactate trafficking inhibition restores sensitivity to proteasome inhibitors and orchestrates immuno-microenvironment in multiple myeloma. Cell Prolif..

[107] Guan X., Rodriguez-Cruz V., Morris M.E. (2019). Cellular Uptake of MCT1 Inhibitors AR-C155858 and AZD3965 and Their Effects on MCT-Mediated Transport of L-Lactate in Murine 4T1 Breast Tumor Cancer Cells. AAPS J..

[108] Goldberg F.W., Kettle J.G., Lamont G.M., Buttar D., Ting A.K.T., McGuire T.M., Cook C.R., Beattie D., Morentin Gutierrez P., Kavanagh S.L. (2022). Discovery of Clinical Candidate AZD0095, a Selective Inhibitor of Monocarboxylate Transporter 4 (MCT4) for Oncology. J. Med. Chem..

[109] Sharma S., Goreczny G., Noonepalle S.K., Palmer E., Garcia-Hernandez M., Banerjee D., Escobedo J., Villagra A., Sandanayaka V. (2021). Abstract 1268: A novel treatment approach for melanoma by dually targeting MCT1 and MCT4 lactate transporters. Cancer Res..

[110] Goreczny G.J., Escobedo J., Sandanayaka V. (2021). Abstract 1335: Dual MCT1/4 inhibition promotes anti-tumor immunity in triple-negative breast cancer. Cancer Res..

[111] Singh M., Afonso J., Sharma D., Gupta R., Kumar V., Rani R., Baltazar F., Kumar V. (2023). Targeting monocarboxylate transporters (MCTs) in cancer: How close are we to the clinics?. Semin. Cancer Biol..

[112] Duan Q., Zhang S., Wang Y., Lu D., Sun Y., Wu Y. (2022). Proton-coupled monocarboxylate transporters in cancer: From metabolic crosstalk, immunosuppression and anti-apoptosis to clinical applications. Front. Cell Dev. Biol..

[113] Yang J., Davis T., Kazerouni A.S., Chen Y.I., Bloom M.J., Yeh H.C., Yankeelov T.E., Virostko J. (2022). Longitudinal FRET Imaging of Glucose and Lactate Dynamics and Response to Therapy in Breast Cancer Cells. Mol. Imaging Biol..

[114] Liu H., Wang S., Wang J., Guo X., Song Y., Fu K., Gao Z., Liu D., He W., Yang L.-L. (2025). Energy metabolism in health and diseases. Signal Transduct. Target. Ther..

[115] Alhusban A.A., Hamadneh L.A., Albustanji S., Shallan A.I. (2022). Lactate and pyruvate levels correlation with lactate dehydrogenase gene expression and glucose consumption in Tamoxifen-resistant MCF-7 cells using capillary electrophoresis with contactless conductivity detection (CE-C^4^D). Electrophoresis.

[116] Yin R., Prabhakaran V., Laskin J. (2018). Quantitative Extraction and Mass Spectrometry Analysis at a Single-Cell Level. Anal. Chem..

[117] Kaur B., Kumar S., Kaushik B.K. (2022). Recent advancements in optical biosensors for cancer detection. Biosens. Bioelectron..

[118] Jiang S., Zhang Y., Yang Y., Huang Y., Ma G., Luo Y., Huang P., Lin J. (2019). Glucose Oxidase-Instructed Fluorescence Amplification Strategy for Intracellular Glucose Detection. ACS Appl. Mater. Interfaces.

[119] Koo K.-M., Kim C.-D., Kim T.-H. (2024). Recent Advances in Electrochemical Detection of Cell Energy Metabolism. Biosensors.

[120] Noreen S., Ishaq I., Saleem M.H., Ali B., Muhammad Ali S., Iqbal J. (2025). Electrochemical biosensing in oncology: A review advancements and prospects for cancer diagnosis. Cancer Biol. Ther..

[121] Cui F., Zhou Z., Zhou H.S. (2020). Review—Measurement and Analysis of Cancer Biomarkers Based on Electrochemical Biosensors. J. Electrochem. Soc..

[122] Baranwal J., Barse B., Gatto G., Broncova G., Kumar A. (2022). Electrochemical Sensors and Their Applications: A Review. Chemosensors.

[123] Sumitha M.S., Xavier T.S. (2023). Recent advances in electrochemical biosensors—A brief review. Hybrid. Adv..

[124] Zahirinejad S., Hemmati R., Homaei A., Dinari A., Hosseinkhani S., Mohammadi S., Vianello F. (2021). Nano-organic supports for enzyme immobilization: Scopes and perspectives. Colloids Surf. B Biointerfaces.

[125] De Zio S., Becconi M., Solda A., Malferrari M., Lesch A., Rapino S. (2023). Glucose micro-biosensor for scanning electrochemical microscopy characterization of cellular metabolism in hypoxic microenvironments. Bioelectrochemistry.

[126] Ma Z., Luo Y., Zhu Q., Jiang M., Pan M., Xie T., Huang X., Chen D. (2020). In-situ monitoring of glucose metabolism in cancer cell microenvironments based on hollow fiber structure. Biosens. Bioelectron..

[127] Lee W.-C., Gurudatt N.G., Park D.-S., Kim K.B., Choi C.S., Shim Y.-B. (2020). Microneedle array sensor for monitoring glucose in single cell using glucose oxidase-bonded polyterthiophene coated on AuZn oxide layer. Sens. Actuators B Chem..

[128] Ding L., Zhao M., Fan S., Ma Y., Liang J., Wang X., Song Y., Chen S. (2016). Preparing Co_3_O_4_ urchin-like hollow microspheres self-supporting architecture for improved glucose biosensing performance. Sens. Actuators B Chem..

[129] Wang C., Zhang X., Liu Y., Li J., Zhu L., Lu Y., Guo X., Chen D. (2022). An enzyme-particle hybrid ink for one step screen-printing and long-term metabolism monitoring. Anal. Chim. Acta.

[130] Lin J., Yuan P., Lin R., Xue X., Chen M., Xing L. (2024). A Self-Powered Lactate Sensor Based on the Piezoelectric Effect for Assessing Tumor Development. Sensors.

[131] Liu D.-M., Dong C. (2020). Recent advances in nano-carrier immobilized enzymes and their applications. Process Biochem..

[132] Wang X., Ma Y., Zhao M., Zhou M., Xiao Y., Sun Z., Tong L. (2016). Determination of glucose in human stomach cancer cell extracts and single cells by capillary electrophoresis with a micro-biosensor. J. Chromatogr. A.

[133] Yao C.-Y., Qin Y., Fan W.-T., Yan L.-P., Chen M., Liu Y.-L., Huang W.-H. (2022). A three-dimensional electrochemical biosensor integrated with hydrogel for cells culture and lactate release monitoring. J. Electroanal. Chem..

[134] Mi S., Xia J., Xu Y., Du Z., Sun W. (2019). An integrated microchannel biosensor platform to analyse low density lactate metabolism in HepG2 cells in vitro. RSC Adv..

[135] Bi Y., Ye L., Mao Y., Wang L., Qu H., Liu J., Zheng L. (2019). Porous carbon supported nanoceria derived from one step in situ pyrolysis of Jerusalem artichoke stalk for functionalization of solution-gated graphene transistors for real-time detection of lactic acid from cancer cell metabolism. Biosens. Bioelectron..

[136] Qi M., Zhang Y., Cao C., Lu Y., Liu G. (2016). Increased sensitivity of extracellular glucose monitoring based on AuNP decorated GO nanocomposites. RSC Adv..

[137] Marquitan M., Ruff A., Bramini M., Herlitze S., Mark M.D., Schuhmann W. (2020). Polymer/enzyme-modified HF-etched carbon nanoelectrodes for single-cell analysis. Bioelectrochemistry.

[138] Nascimento R.A., Ozel R.E., Mak W.H., Mulato M., Singaram B., Pourmand N. (2016). Single Cell “Glucose Nanosensor” Verifies Elevated Glucose Levels in Individual Cancer Cells. Nano Lett..

[139] Madhurantakam S., Jayanth Babu K., Balaguru Rayappan J.B., Krishnan U.M. (2017). Fabrication of mediator-free hybrid nano-interfaced electrochemical biosensor for monitoring cancer cell proliferation. Biosens. Bioelectron..

[140] Ali M., Khalid M.A.U., Kim Y.S., Soomro A.M., Hussain S., Doh Y.H., Choi K.H. (2021). MWCNTs/PEDOT: PSS Composite as Guiding Layer on Screen-Printed Carbon Electrode for Linear Range Lactate Detection. J. Electrochem. Soc..

[141] Hashemzadeh S., Omidi Y., Rafii-Tabar H. (2019). Amperometric lactate nanobiosensor based on reduced graphene oxide, carbon nanotube and gold nanoparticle nanocomposite. Mikrochim. Acta.

[142] Wu H., Zhang X., Wei C., Wang C., Jiang M., Hong X., Xu Z., Chen D., Huang X. (2021). Modular assembly of enzyme loaded nanoparticles in 3D hollow fiber electrode for electrochemical sensing. Chem. Eng. J..

[143] Malik M., Chaudhary R., Pundir C.S. (2020). An amperometric pyruvate biosensor based on pyruvate oxidase nanoparticles immobilized onto pencil graphite electrode. Process Biochem..

[144] Malik M., Chaudhary R., Pundir C.S. (2019). An improved enzyme nanoparticles based amperometric pyruvate biosensor for detection of pyruvate in serum. Enzym. Microb. Technol..

[145] Kucherenko I.S., Soldatkin O.O., Topolnikova Y.V., Dzyadevych S.V., Soldatkin A.P. (2019). Novel Multiplexed Biosensor System for the Determination of Lactate and Pyruvate in Blood Serum. Electroanalysis.

[146] Bilal M., Khaliq N., Ashraf M., Hussain N., Baqar Z., Zdarta J., Jesionowski T., Iqbal H.M.N. (2023). Enzyme mimic nanomaterials as nanozymes with catalytic attributes. Colloids Surf. B Biointerfaces.

[147] Hwang D.-W., Lee S., Seo M., Chung T.D. (2018). Recent advances in electrochemical non-enzymatic glucose sensors—A review. Anal. Chim. Acta.

[148] Wei M., Qiao Y., Zhao H., Liang J., Li T., Luo Y., Lu S., Shi X., Lu W., Sun X. (2020). Electrochemical non-enzymatic glucose sensors: Recent progress and perspectives. Chem. Commun..

[149] Saputra H.A. (2023). Electrochemical sensors: Basic principles, engineering, and state of the art. Monatsh. Chem..

[150] Hassan M.H., Vyas C., Grieve B., Bartolo P. (2021). Recent Advances in Enzymatic and Non-Enzymatic Electrochemical Glucose Sensing. Sensors.

[151] Kader M.A., Suhaity Azmi N., Kafi A.K.M. (2023). Recent advances in gold nanoparticles modified electrodes in electrochemical nonenzymatic sensing of chemical and biological compounds. Inorg. Chem. Commun..

[152] Utagawa Y., Ino K., Shinoda Y., Yamazaki M., Abe H., Shiku H. (2024). Enzyme-Free In-Situ Electrochemical Measurement Using a Porous Membrane Electrode for Glucose Transport into Cell Spheroids. ACS Sens..

[153] Li Q., Zhang Y., Fan H., Gong Y., Xu Y., Lv Q., Xu Y., Xiao F., Wang S., Wang Z. (2021). In vitro and in vivo detection of lactate with nanohybrid-functionalized Pt microelectrode facilitating assessment of tumor development. Biosens. Bioelectron..

[154] Wang W., Mao Z., Lan X., Tian D., Peng J., Chen Y. (2025). Non-enzymatic monitoring of organoid culture media using a microfluidic device with screen-printed electrodes. Mater. Res. Bull..

[155] Grasso G., Colella F., Forciniti S., Onesto V., Iuele H., Siciliano A.C., Carnevali F., Chandra A., Gigli G., Del Mercato L.L. (2023). Fluorescent nano- and microparticles for sensing cellular microenvironment: Past, present and future applications. Nanoscale Adv..

[156] Ma J., Sun R., Xia K., Xia Q., Liu Y., Zhang X. (2024). Design and Application of Fluorescent Probes to Detect Cellular Physical Microenvironments. Chem. Rev..

[157] Li X.F., Wu F.G. (2025). Aggregation-induced emission-based fluorescent probes for cellular microenvironment detection. Biosens. Bioelectron..

[158] Yao L., Yin C., Huo F. (2022). Small-Molecule Fluorescent Probes for Detecting Several Abnormally Expressed Substances in Tumors. Micromachines.

[159] Jun J.V., Chenoweth D.M., Petersson E.J. (2020). Rational design of small molecule fluorescent probes for biological applications. Org. Biomol. Chem..

[160] Wu L., Liu J., Li P., Tang B., James T.D. (2021). Two-photon small-molecule fluorescence-based agents for sensing, imaging, and therapy within biological systems. Chem. Soc. Rev..

[161] Liang Z., Pang H., Zeng G., Chen T. (2022). Bioorthogonal Light-Up Fluorescent Probe Enables Wash-Free Real-Time Dynamic Monitoring of Cellular Glucose Uptake. Anal. Chem..

[162] Cheng Y., Shabir G., Li X., Fang L., Xu L., Zhang H., Li E. (2020). Development of a deep-red fluorescent glucose-conjugated bioprobe for in vivo tumor targeting. Chem. Commun..

[163] Jo A., Sung J., Lee S., Nam H., Lee H.W., Park J., Kim H.M., Kim E., Park S.B. (2018). Near-IR Fluorescent Tracer for Glucose-Uptake Monitoring in Live Cells. Bioconjug. Chem..

[164] Wang K., Zhang R., Zhao X., Ma Y., Ren L., Ren Y., Chen G., Ye D., Wu J., Hu X. (2023). Reversible Recognition-Based Boronic Acid Probes for Glucose Detection in Live Cells and Zebrafish. J. Am. Chem. Soc..

[165] Li M., Li X., Chen L., Li X., Liu C. (2025). An “off-on” fluorescent probe for imaging pyruvic acid in living systems. Talanta.

[166] Rajalakshmi K., Muthusamy S., Nam Y.S., Li Y., Lee K.B., Xu Y. (2022). A new recognition moiety diphenylborinate in the detection of pyruvate via Lewis acid/base sensing pathway and its bioimaging applications. Spectrochim. Acta A Mol. Biomol. Spectrosc..

[167] Zhang X., Ding S., Cao S., Zhu A., Shi G. (2016). Functional surface engineering of quantum dot hydrogels for selective fluorescence imaging of extracellular lactate release. Biosens. Bioelectron..

[168] Gao Y., Wang Y., He B., Pan Y., Zhou D., Xiong M., Song Y. (2023). An Enzyme-Loaded Metal-Organic Framework-Assisted Microfluidic Platform Enables Single-Cell Metabolite Analysis. Angew. Chem. Int. Ed. Engl..

[169] Sargazi S., Fatima I., Hassan Kiani M., Mohammadzadeh V., Arshad R., Bilal M., Rahdar A., Diez-Pascual A.M., Behzadmehr R. (2022). Fluorescent-based nanosensors for selective detection of a wide range of biological macromolecules: A comprehensive review. Int. J. Biol. Macromol..

[170] Mohammadi R., Naderi-Manesh H., Farzin L., Vaezi Z., Ayarri N., Samandari L., Shamsipur M. (2022). Fluorescence sensing and imaging with carbon-based quantum dots for early diagnosis of cancer: A review. J. Pharm. Biomed. Anal..

[171] Henrique R.B.L., Santos A.L.F., Pereira M.I.A., Oliveira W.F., Santos B.S., Pereira G., Fontes A., Cabral Filho P.E. (2023). A fluorescent glyconanoprobe based on quantum dots and thiolated glucose: Applications in monolayers and spheroids of cancer cells. Biochim. Biophys. Acta Gen. Subj..

[172] Ripoll C., Orte A., Paniza L., Ruedas-Rama M.J. (2019). A Quantum Dot-Based FLIM Glucose Nanosensor. Sensors.

[173] Sun Y., Shu T., Ma J., Dai Q., Peng P., Zhou Z., Zhou X., Su L., Zhang X. (2022). Rational Design of ZIF-8 for Constructing Luminescent Biosensors with Glucose Oxidase and AIE-Type Gold Nanoclusters. Anal. Chem..

[174] Zhang Y., Xu L., Ge J. (2022). Multienzyme System in Amorphous Metal-Organic Frameworks for Intracellular Lactate Detection. Nano Lett..

[175] Zou Y., Wang A., Shi M., Chen X., Liu R., Li T., Zhang C., Zhang Z., Zhu L., Ju Z. (2018). Analysis of redox landscapes and dynamics in living cells and in vivo using genetically encoded fluorescent sensors. Nat. Protoc..

[176] Zhang Z., Cheng X., Zhao Y., Yang Y. (2020). Lighting Up Live-Cell and In Vivo Central Carbon Metabolism with Genetically Encoded Fluorescent Sensors. Annu. Rev. Anal. Chem..

[177] Hario S., Le G.N.T., Sugimoto H., Takahashi-Yamashiro K., Nishinami S., Toda H., Li S., Marvin J.S., Kuroda S., Drobizhev M. (2024). High-Performance Genetically Encoded Green Fluorescent Biosensors for Intracellular l-Lactate. ACS Cent. Sci..

[178] Nasu Y., Murphy-Royal C., Wen Y., Haidey J.N., Molina R.S., Aggarwal A., Zhang S., Kamijo Y., Paquet M.E., Podgorski K. (2021). A genetically encoded fluorescent biosensor for extracellular L-lactate. Nat. Commun..

[179] Nasu Y., Aggarwal A., Le G.N.T., Vo C.T., Kambe Y., Wang X., Beinlich F.R.M., Lee A.B., Ram T.R., Wang F. (2023). Lactate biosensors for spectrally and spatially multiplexed fluorescence imaging. Nat. Commun..

[180] Li X., Zhang Y., Xu L., Wang A., Zou Y., Li T., Huang L., Chen W., Liu S., Jiang K. (2023). Ultrasensitive sensors reveal the spatiotemporal landscape of lactate metabolism in physiology and disease. Cell Metab..

[181] Ruiz-Rodado V., Lita A., Larion M. (2022). Advances in measuring cancer cell metabolism with subcellular resolution. Nat. Methods.

